# Supplementary material for: Content-rich biological network constructed by mining PubMed abstracts
Source: BMC Bioinformatics. 2004 Oct 8;5:147. doi: 10.1186/1471-2105-5-147 (PMC528731; doi:10.1186/1471-2105-5-147)
Supplement: Additional File 2 — The original results of the above study (non-essential files are deleted to keep the file size under the limit set by BMC bioinformatics). [file 1471-2105-5-147-S2.bz2 › chilibotAdditionalFile2/dip05/66ID7729427E226/html/TAF11_TAF12.html]

 


 **TAF11** and **TAF12** 
  
Found 1 abstracts in PubMed, retrieved 1.  
 

 What does Google say? 
 PDF only 
| .edu only 

---

**Interactive relationship** (e.g. stimulation, inhibition, etc)

**Inhibitory relationship**- Mutations in the histone fold domain of the  **TAF12**  gene show synthetic lethality with the TAF1  [ **TAF11** ]  gene lacking the TAF N terminal domain TAND by different mechanisms from those in the SPT15 gene encoding the TATA box binding protein TBP.  Ref: 12582246 Nucleic Acids Res, 2003
**Neutral relationship**- Interestingly, a set of genes affected in the taf1  [ **TAF11** ]  DeltaTAND mutant is similarly affected in the  **taf12**  HFD mutants but not in the nsl mutants of TBP.  Ref: 12582246 Nucleic Acids Res, 2003
